# Significance of predicted future liver remnant volume on liver failure risk after major hepatectomy: a case matched comparative study

**DOI:** 10.3389/fsurg.2023.1174024

**Published:** 2023-05-17

**Authors:** R. Piccus, K. Joshi, J. Hodson, D. Bartlett, N. Chatzizacharias, B. Dasari, J. Isaac, R. Marudanayagam, D. F. Mirza, J. K. Roberts, R. P. Sutcliffe

**Affiliations:** University of Birmingham, Birmingham, United Kingdom; Liver Unit, Queen Elizabeth Hospital, Birmingham, United Kingdom; Institute of Translational Medicine, Queen Elizabeth Hospital, Birmingham, United Kingdom; Department of Health Informatics, Queen Elizabeth Hospital, Birmingham, United Kingdom

**Keywords:** hepatectomy, post-hepatectomy liver failure, volumetric analysis, liver volumetry, major hepatectomy

## Abstract

**Introduction:**

Future liver remnant volume (FLRV), a risk factor for liver failure (PHLF) after major hepatectomy (MH), is not routinely measured. This study aimed to evaluate the association between FLRV and PHLF.

**Patients and methods:**

All patients undergoing MH (4 + segments) between 2011 and 2018 were identified from a prospectively maintained single-centre database. Perioperative data were collected for patients with PHLF, who were matched (1:2) with non-PHLF controls. FLRV and FLRV_%_ (i.e., % of total liver volume) were calculated retrospectively from preoperative CT scans using Synapse-3D software, and compared between the PHLF and matched control groups.

**Results:**

Of 711 patients undergoing MH, PHLF occurred in 27 (3.8%), of whom 24 had preoperative CT scans available. These patients were matched to 48 non-PHLF controls, 98% of whom were classified as being at high risk of PHLF on preoperative risk scoring. FLRV_%_ was significantly lower in the PHLF group, compared to matched controls (median: 28.7 vs. 35.2%, *p* = 0.010), with FLRV% < 30% in 58% and 29% of patients, respectively. Assessment of the ability of FLRV_%_ to differentiate between PHLF and matched controls returned an area under the ROC curve of 0.69, and an optimal cut-off value of FLRV_%_ < 31.5%, which yielded 79% sensitivity and 67% specificity.

**Conclusions:**

FLRV_%_ is significantly predictive of PHLF after MH, with over half of patients with PHLF having FLRV_%_ < 30%. In light of this, we propose that all patients should undergo risk stratification prior to MH, with the high risk patients additionally being assessed with CT volumetry.

## Introduction

Post-hepatectomy liver failure (PHLF) is an uncommon but potentially fatal complication after major hepatectomy (1, 2). The risk factors for PHLF are well established, and include patient demographics and the presence/severity of underlying liver disease (3–5). The importance of an adequate remnant liver volume has been recognised for several decades (6), and a minimum threshold future liver remnant volume (FLRV) of 20%–25% of the total liver volume (TLV) had been proposed, based on historical studies of patients with normal livers (7–9). Although liver volumetric software programs are available, preoperative volumetry is not universally undertaken prior to major hepatectomy, due to high cost. Whilst patients who are being considered for extended hepatectomy often undergo formal volumetric analysis, the adequacy of the FLRV before right hemihepatectomy is frequently determined by subjective assessment of the FLRV on preoperative imaging, due to a perceived low risk of PHLF in this group. Although the average left liver lobe volume is approximately 40% of TLV, there is a wide range of values (18%–51%) (10, 11). A significant proportion of patients have a left lobe volume less than 30% of TLV (12, 13), and the incidence of PHLF after right hemihepatectomy may be as high as 5%, based on a study of healthy live liver donors (14). The primary aim of this study was to evaluate the association between volumetric analysis and PHLF after major hepatectomy.

## Patients and methods

### Case selection

All patients who underwent a major hepatectomy (defined as resection of four or more segments) in a single UK tertiary hepatobiliary unit between January 2011 and December 2018 were retrospectively identified from a prospectively maintained database. Exclusion criteria were patients who underwent two-stage hepatectomy (including ALPPS) or preoperative portal vein embolization (*N* = 76). The group of patients developing PHLF were then identified from the database, with severity of PHLF being classified according to the ISGLS criteria (15). Patients with PHLF were then matched 1:2 to patients without PHLF. Matching was performed using an *ad hoc* approach, with patients being matched on age, gender, type of resection (right, extended right, or extended left hemihepatectomy), indication for surgery, and preoperative chemotherapy status. Patients were exact-matched on all factors, with the exception of age, where the control case with the closest age to each PHLF case was used. In addition, it was not possible to find matches for the indication for surgery for three PHLF patients; hence this factor was disregarded when matching these cases, in order to prevent exclusions. For the matched cohorts, data for additional demographics, as well as peri- and postoperative factors were collected, with the expected risk of PHLF being quantified using the preoperative PHLF risk score (16).

### CT volumetric analysis

During the study period, for patients undergoing extended (left or right) hepatectomy, CT volumetric analysis was performed selectively in cases where it was felt to be warranted, based on the surgeon's subjective assessment of the future liver remnant (FLR). Preoperative CT volumetric analysis was not routinely undertaken in patients undergoing right hemihepatectomy. As such, for patients in the PHLF and matched control groups, liver volumetric analysis was carried out retrospectively from preoperative CT scans, using Fujifilm Synapse imaging software (Fujifilm, Japan). From this, the right (RLV) and left liver lobe volumes (LLV) were calculated, both as absolute values and as a proportion of TLV [e.g., RLV_% _= 100*(RLV/TLV)]. The volume to be resected was then calculated, and used to estimate FLRV. Again, this was assessed as both an absolute value, and as a percentage of TLV [FLRV_%_ = 100*(FLRV/TLV)]. The FLRV_%_ was deemed to be “inadequate” if <30%, “borderline” if 30%–39% and “adequate” if ≥40%.

### Statistical methods

Initially, the control patients included in the matched cohort were compared to those not selected by the matching procedure, in order to assess how the matched cohort compared to the population as a whole. Within the matched cohort, comparisons were then made between PHLF and control patients, to test whether these groups were comparable at baseline. In each case, ordinal or continuous variables were compared using Mann-Whitney U tests and summarised using the mean ± standard deviation (SD) where approximately normally distributed, with the median and interquartile range (IQR) reported otherwise. Nominal variables were analysed using Fisher's exact tests. The ability of CT volumetry data to differentiate between the PHLF and matched control groups was then assessed using receiver operating characteristic (ROC) curves, and quantified using the area under the curve (AUROC). A binary logistic regression model was also produced, with the FLRV_%_ as a continuous covariate, to produce an odds ratio. The optimum cut-off value of FLRV_%_ for the discrimination between the PHLF and matched control groups was then estimated, based on the value with the highest Youden's J statistic. All analyses were performed using IBM SPSS 24 (IBM Corp. Armonk, NY), with *p* < 0.05 deemed to be indicative of statistical significance throughout.

## Results

### Overall cohort characteristics

A total of 711 patients underwent major hepatectomy during the study period, including 476 right hemihepatectomies (RH) and 235 extended hemihepatectomies (EH; comprising 152 extended right, and 83 extended left). Of the entire cohort, 27 patients (3.8%) developed PHLF, with 5, 11 and 11 of grade A, B and C, respectively. The incidence of PHLF after RH and EH was 12/476 (2.5%) and 15/235 (6.4%), respectively. There were six PHLF-related deaths, including two deaths after RH.

### Liver volumetry in patients developing PHLF

Preoperative CT scans were unavailable for 3/27 (11%) patients with PHLF; hence retrospective CT liver volumetric analysis could only be performed in the remaining 24 cases. The demographics of this group are reported in Table 1, with liver volumetry reported in Table 2. These patients had a median TLV of 1,707 ml (IQR: 1,361–2,154), and median left and right lobe volumes of 656 ml (IQR: 499–848) and 932 ml (IQR: 839–1,312), respectively. The left lobe volume was a median of 37.1% (IQR: 31.5–43.6) of TLV, and three patients (27%) who developed PHLF after RH had a left lobe volume of <30% of TLV. The median FLRV_%_ in patients undergoing RH (30.6%, IQR: 28.2–37.0) was significantly higher than in those undergoing EH (26.5%, IQR: 22.6–29.3; *p* = 0.014), with FLRV_%_ being <30% in 4/11 (36%) and 10/13 (77%) patients who developed PHLF after RH and EH, respectively. Three patients developed PHLF despite having FLRV_%_ ≥ 40%, including two patients who developed grade B PHLF after right hemihepatectomy and had radiological drainage of intra-abdominal collections. The third patient developed severe venous congestion of the liver remnant following an extended left hemihepatectomy with significant intraoperative blood loss, and subsequently developed multi-organ failure and died on the third postoperative day.

**Table 1:** Cohort characteristics.

	Matched					Unmatched		
	PHLF		Control		*p*-Value^a^	Control		*p*-Value^b^
	*N*	Statistic	*N*	Statistic		*N*	Statistic	
Age (years)	24	65.2 ± 8.1	48	65.5 ± 8.3	0.816	630	62.9 ± 12.4	0.267
Gender (% male)	24	18 (75%)	48	36 (75%)	*N/A*	630	338 (54%)	**0**.**004**
BMI (kg/m^2^)	24	26.8 ± 4.6	47	28.2 ± 4.3	0.193	560	28.0 ± 4.9	0.856
Liver disease	24		48		0.896			–
None		13 (54%)		29 (60%)		–	–	
Post-chemotherapy		5 (21%)		8 (17%)		–	–	
Cholestasis		4 (17%)		8 (17%)		–	–	
Steatosis/steatohepatitis		2 (8%)		2 (4%)		–	–	
Fibrosis/cirrhosis		0 (0%)		1 (2%)		–	–	
Myocardial infarction	24	3 (13%)	48	2 (4%)	0.325	–	–	–
Diabetes mellitus	24	5 (21%)	48	4 (8%)	0.149	–	–	–
Charlson comorbidity index	24	9 (5–9)	48	8 (5–9)	0.655	–	–	–
Indication for surgery	24		48		0.663	630		**<0**.**001**
Colorectal metastases		11 (46%)		25 (52%)			407 (65%)	
Cholangiocarcinoma		8 (33%)		17 (35%)			53 (8%)	
Hepatocellular carcinoma		0 (0%)		0 (0%)			47 (7%)	
Others		5 (21%)		6 (13%)			123 (20%)	
Preoperative chemotherapy^d^	24	9 (38%)	48	18 (38%)	*N/A*	–	–	–
PHLF risk score^e^	24	10.5 (9.3–11.0)	48	10.3 (9.0–10.5)	0.247	–	–	–
Type of surgery	24		48		*N/A*	630		**<0** **.** **001**
Right hemihepatectomy		11 (46%)		22 (46%)			440 (70%)	
Extended right hemihepatectomy		11 (46%)		22 (46%)			113 (18%)	
Extended left hemihepatectomy		2 (8%)		4 (8%)			77 (12%)	
Vascular resection	24	3 (13%)	48	9 (19%)	0.739	–	–	–
Inflow occlusion	24	7 (29%)	48	16 (33%)	0.794	–	–	–
Perioperative blood transfusion	21		48		**0**.**005**^c^	624		0.432^c^
None		11 (52%)		41 (85%)			503 (81%)	
1–2 units		7 (33%)		4 (8%)			76 (12%)	
* >2 units*		3 (14%)		3 (6%)			45 (7%)	
Postoperative complications								
Bile leak/intra-abdominal sepsis	24	2 (8%)	48	7 (15%)	0.708	–	–	–
Portal vein thrombosis	24	1 (4%)	48	0 (0%)	0.333	–	–	–
Radiological drainage of fluid collections	24	11 (46%)	48	5 (10%)	0.002	–	–	–

Data were only collected for selected variables in the unmatched control cohort. Ordinal and continuous variables are reported as mean ± standard deviation, or as median (interquartile range), with *p*-values from Mann-Whitney *U* tests. Nominal variables are reported as *N* (column %), with *p*-values from Fisher's exact tests, unless stated otherwise. Bold *p*-values are significant at *p* < 0.05. N/A = not applicable, as patients were exact-matched on the stated factor.

^a^ *p*-Values comparing the PHLF vs. control groups in the matched cohort.

^b^ *p*-Values comparing the control groups in the matched vs. unmatched cohorts.

^c^ *p*-Value from Mann-Whitney *U* test, as the factor is ordinal.

^d^ With Oxaliplatin or Irinotecan.

^e^ Reference (16).

**Table 2:** Volumetric analysis.

	PHLF (*N* = 24)	Matched control (*N* = 48)	AUROC (95% CI)^a^	*p*-Value
Left liver volume (ml)	656 (499–848)	677 (543–822)	0.55 (0.40–0.70)^b^	0.488
Left liver volume (%)	37.1 (31.5–43.6)	41.6 (36.7–47.0)	0.66 (0.52–0.80)^b,c^	**0.025** ^c^
Right liver volume (ml)	932 (839–1,312)	911 (764–1,179)	0.60 (0.47–0.74)	0.152
Right liver volume (%)	62.9 (56.4–68.5)	58.4 (53.1–63.4)	0.66 (0.52–0.80)^c^	**0.025** ^c^
Total liver volume (ml)	1,707 (1,361–2,154)	1,595 (1,393–1,942)	0.53 (0.38–0.68)	0.685
Volume resected (ml)	1,175 (964–1,442)	1,079 (864–1,265)	0.60 (0.46–0.74)	0.164
FLRV (ml)	486 (399–590)	546 (439–687)	0.61 (0.47–0.76)^b^	0.116
FLRV_%_ (%)	28.7 (26.1–31.2)	35.2 (27.9–39.4)	0.69 (0.56–0.82)^b^	**0**.**010**
<30% (inadequate)	14 (58%)	14 (29%)		
30–39% (borderline)	7 (29%)	23 (48%)		
≥40% (adequate)	3 (13%)	11 (23%)		

Data are reported as median (interquartile range), or as *N* (%), with *p*-values from Mann-Whitney *U* tests. Bold *p*-values are significant at *p* < 0.05.

^a^ Area under the receiver operating characteristic curve (AUROC); AUROCs > 0.5 indicate factors where values were higher in those with PHLF, unless stated otherwise.

^b^ Values were lower in those with PHLF.

^c^ Since the left liver volume as a percentage of the total is the inverse of the right, the AUROCs and *p*-values are identical for both factors.

### Selection of matched controls

The 27 patients with PHLF were then matched in a 1:2 ratio to 54 control patients without PHLF. After excluding those for whom preoperative CT scans were unavailable, 24 patients with PHLF and 48 patients without PHLF were included in subsequent analysis. Comparisons between the matched controls and 630 unmatched controls found those included in the matched analysis to be significantly more likely to be male (75% vs. 54%, *p* = 0.004), to have cholangiocarcinoma (35% vs. 8%, *p* < 0.001) and to have undergone extended right hemihepatectomy (46% vs. 18%, *p* < 0.001) than the remainder of the non-PHLF group (Table 1). As such, the matched controls were identified as being a biased sample of the cohort as a whole with very high risk of PHLF, as would be expected given the matching procedure; the median preoperative risk score for matched controls was 10.3 (IQR: 9.0–10.5), and 98% (47/48) had risk scores above the proposed high-risk threshold of >5.5(16).

### Comparisons between patients developing PHLF and matched controls

Comparisons between the PHLF and matched control groups found these to have similar baseline characteristics, including similar PHLF risk scores (median: 10.5 vs. 10.3, *p* = 0.247); 38% of patients in each cohort received chemotherapy prior to hepatectomy (Table 1). Analysis of liver volumetry (Table 2) found the TLV to be similar in the PHLF and matched control groups (median: 1,707 vs. 1,595 ml, *p* = 0.685). However, the left liver volume was a significantly smaller percentage of TLV in those developing PHLF, compared to matched controls (median: 37.1 vs. 41.6%, *p* = 0.025).

The average FLRV_%_ was significantly lower in the PHLF group compared to matched controls (median 28.7 vs. 35.2%; *p* = 0.010), with 58% vs. 29% having FLRV_%_ < 30%. The associated AUROC for the differentiation between the PHLF and matched control groups was 0.69 (95% CI: 0.56–0.82), with binary logistic regression returning an odds ratio of 0.92 (95% CI: 0.86–0.99) per unit increase in FLRV_%_. Based on the Youden's J statistic, the optimum cut-off value of FLRV_%_ for discriminating between the PHLF and matched control groups was 31.5%. Of those with FLRV_%_ < 31.5%, 19/35 (54%) were in the PHLF group, compared to 5/37 (14%) of those with FLRV_%_ ≥ 31.5%, yielding 79% sensitivity and 67% specificity.

### Perioperative factors and postoperative outcomes

The proportions of patients who underwent a concomitant vascular resection or had inflow occlusion were similar in the PHLF and matched control groups (Table 1). The incidence of postoperative bile leak, intra-abdominal sepsis and portal vein thrombosis were also similar between groups. However, patients developing PHLF were significantly more likely to receive a perioperative blood transfusion (48% vs. 15%; *p* = 0.005) and to require radiological drainage of intra-abdominal collections (46% vs. 10%; *p* = 0.002) than matched controls.

## Discussion

The importance of the adequacy of the future liver remnant volume to minimize the risk of PHLF after major hepatectomy is well established (17). However, liver volumetric analysis is not routinely performed prior to major hepatectomy in many centres, due to cost and/or limited resources. The primary aim of this study was to assess the relationship between liver volumetric analysis and PHLF. This would be useful in determining whether routine utilisation of preoperative volumetry analysis in treatment planning could have the potential to reduce the incidence of PHLF.

The most notable finding of this study is that more than half of patients who developed PHLF in this series had an “inadequate” FLRV_%_ of <30%, including one third of patients who developed PHLF after RH. All 14 patients with FLRV_%_ < 30% who developed PHLF were classified as high-risk based on preoperative risk scoring (scores >5.5). As such, if preoperative volumetric analysis had been undertaken for all high-risk patients in this series, then treatment plans could have been modified in these patients, in order to minimise the risk of PHLF. For example, these patients could have been referred for preoperative portal/hepatic vein embolization to induce hypertrophy of the liver remnant, or may have been considered for non-surgical therapies. Because PHLF has no effective treatment, management is aimed at multi-organ support and is associated with significant morbidity, mortality and healthcare costs (18). Prevention of PHLF is therefore essential, and based on data from this study, preoperative volumetric analysis can be justified in high-risk patients prior to major hepatectomy, including right hemihepatectomy.

Whilst the majority of patients developing PHLF had an “inadequate” FLRV_%_, PHLF also developed in seven patients with a “borderline” FLRV_%_ (30%–39%) and in three patients with an “adequate” FLRV_%_ (≥40%). This may be, in part, due to the threshold used for “inadequate” FLRV_%_, with the optimum cut-off in our analysis being higher, at 31.5%. In addition, in patients with borderline or adequate FLR, the mechanism of PHLF is likely to be multifactorial, including impaired global liver function and/or postoperative complications (e.g., haemorrhage, vascular thrombosis or sepsis). A significant proportion of patients undergoing major hepatectomy have parenchymal liver injury (e.g., chemotherapy induced damage, biliary obstruction or steatosis/steatohepatitis) that may predispose patients to impaired postoperative liver function and/or PHLF. Although reversible causes such as biliary obstruction must be corrected prior to surgery, the impact of irreversible causes on global liver function may be quantified preoperatively. The 99^m^ Technetium-Mebrofinate SPECT-CT scan is an emerging modality that provides anatomical and functional assessment of liver function, but is not yet widely available (19). Meticulous surgical technique and prompt diagnosis and treatment of postoperative complications are essential in order to minimize the impact on liver function in patients with borderline FLRV_%_.

In addition to the 14 patients with PHLF with “inadequate” FLRV_%_ on preoperative liver volumetry, there were a further 14 matched controls who did not develop PHLF, despite having FLRV_%_ < 30%. These patients comprised 29% of the matched control cohort, although this is likely a considerable overestimate of the rate of “inadequate” FLRV_%_ in the non-PHLF population as a whole, since the matching procedure selected a highly biased and very high-risk control cohort. Despite this, these patients highlight that use of preoperative liver volumetry in isolation to plan treatment may result in under-treatment in some patients who would not have gone on to develop PHLF. As such, preoperative liver volumetry needs to be utilised alongside other information during the decision-making process.

This study has several limitations. None of the patients with PHLF in our series had underlying cirrhosis, and this is a reflection of the small number of cirrhotic patients who undergo major hepatectomy in our unit. Therefore, the results of this study cannot be applied to this subgroup, and further study of cirrhotic patients would be required. Whilst matching PHLF patients to controls constituted a strength of the study, by negating the effect of potentially confounding factors on the comparisons between groups, it also represented a limitation. The process of matching control patients to those with PHLF resulted in the selection of higher risk control patients, with a greater proportion of males, patients with cholangiocarcinoma, and those undergoing extended surgeries, compared to the controls not included in the study. As such, the cases included in the study are a biased subset of the cohort as a whole. Consequently, if the associations considered in the analysis vary with PHLF risk, then the findings may not be applicable to the cohort of patients undergoing hemihepatectomy as a whole.

In conclusion, the incidence of post-hepatectomy liver failure could potentially be reduced by performing liver volumetric analysis prior to major hepatectomy in high-risk patients. Although the extent of resection is an important factor, a large proportion of cases of PHLF in this series occurred after right hemihepatectomy.

## Data Availability

All data generated or analysed during this study are included in this article. Further enquiries can be directed to the corresponding author.
